# Effect of Beta Cyclodextrin on the Reduction of Cholesterol in Ewe’s Milk Manchego Cheese

**DOI:** 10.3390/molecules23071789

**Published:** 2018-07-20

**Authors:** Leocadio Alonso, Patrick F. Fox, María V. Calvo, Javier Fontecha

**Affiliations:** 1 Instituto de Productos Lácteos de Asturias (CSIC), Paseo Río Linares s/n. 33300 Villaviciosa, Asturias, Spain; 2 School of Food and Nutritional Sciences, University College Cork (UCC), T12 Y337 Cork, Ireland; pff@ucc.ie; 3 Instituto de Investigación en Ciencias de la Alimentación (CSIC-UAM), 28049 Madrid, Spain; mv.calvo@csic.es (M.V.C.); j.fontecha@csic.es (J.F.)

**Keywords:** beta cyclodextrin, ewe’s milk, cheese, Manchego, lipids, cholesterol

## Abstract

Beta-cyclodextrin (β-CD) is a cyclic oligosaccharide consisting of seven glucose units and is produced from starch using cyclodextrin glycotransferase enzymes to break the polysaccharide chain and forming a cyclic polysaccharide molecule. The use of β-CD in food research for reduction of cholesterol is increasing due to its affinity for non-polar molecules such as cholesterol. The aim of this study was to evaluate the feasibility of using β-CD in cholesterol removal from pasteurized ewe’s milk Manchego cheese and evaluate the effect on the main components of the milk, lipids, and flavor characteristics. Approximately 97.6% cholesterol reduction was observed in the cheese that was treated using β-CD. Physicochemical properties (fat, moisture and protein) were not changed by the β-CD treatment, except the soluble nitrogen and non-protein nitrogen that showed slight differences after the treatment. The amount of the different components of the lipid fraction (fatty acids, triglycerides and phospholipids) were similar in cheeses treated and not treated with β-CD. Flavor compound and short chain free fatty acids were not mostly significantly influenced by the effect of the β-CD. β-CD molecules are edible and nontoxic and as a result they can be used safely for cholesterol removal processing in cheese manufacturing. Therefore, the present study suggests that β-CD treatment is an effective process for cholesterol removal from Manchego cheese while preserving its properties.

## 1. Introduction

Although dairy products in general have the image of being healthy foods, this is often not the case for products with a high fat content such as butter, cream and cheeses. The World Health Organization and the American Heart Association have recommended that consumers reduce their consumption of saturated fatty acids and cholesterol to lower the risk of coronary heart disease. This advice, coupled with radical opinions, have created a demand for low-cholesterol products [1]. Nowadays, there is a growing interest in the manufacture of cholesterol-reduced dairy products. Food companies have developed many methods to reduce cholesterol, however, most of these methods are relatively nonselective and remove flavor and nutritional components when cholesterol is removed. Moreover, some methods require high investment and operation costs. Methods for reducing cholesterol in foods have been developed including blending with vegetable oils [2,3], extraction by distillation and crystallization [4,5], adsorption with saponin and digitonin [6,7], assimilation of cholesterol by enzymes from microorganisms [8,9] and removal by supercritical carbon dioxide extraction [10,11]. In the last years, several studies have been published describing the use of β-CD in food applications [12,13,14]. It has been proved that the β-CD molecule can be used as non-toxic and non-digestible molecule to remove cholesterol effectively from milk and dairy products, egg yolk, and lard [15,16,17,18,19,20] with much less investment and operation costs. β-CD is a cyclic oligosaccharide consisting of seven glucose units and is produced from starch using cyclodextrin glycotransferase enzymes, to break the polysaccharide chains and form cyclic polysaccharide molecules. The molecule of β-CD is doughnut shaped and its central portion is a circular hydrophobic space similar in diameter to a cholesterol molecule, giving the molecule its affinity for non-polar molecules such as cholesterol [21,22].

Manchego cheese is one of the most representative of the Spanish hard cheeses. It is manufactured in the region of Castilla-La Mancha (Spain) using pure ewe’s milk from local herds under conditions regulated by an origin appellation. Manchego cheese is a rich in fat (the fat content in the dry cheese is higher than 50%) [23,24], and possesses a characteristic sharp flavor, which increases with the ripening time. Its texture is smooth but consistent, and a few irregular holes randomly distributed in ivory-colored paste. Although the most of investigations for removing cholesterol in milk using β-CD were performed in cow’s milk, no investigations have been reported on the effect of β-CD on reduction of cholesterol in ewe’s milk. Therefore, the aims of this study was to evaluate the feasibility of the β-CD in cholesterol removal from pasteurized ewe’s milk Manchego cheese and its effect on the main components of milk, focusing especially on the lipidic fractions, and flavor characteristics.

## 2. Results and Discussion

### 2.1. Gross Composition

Due to the structural characteristics of β-CD and processing conditions used during cholesterol removal with β-CD, it is possible that some of the milk constituents are also entrapped and removed along with cholesterol. Thus, it is important to investigate the compositional changes occurring during the cholesterol removal process in Manchego cheese.

**Figure 1:**
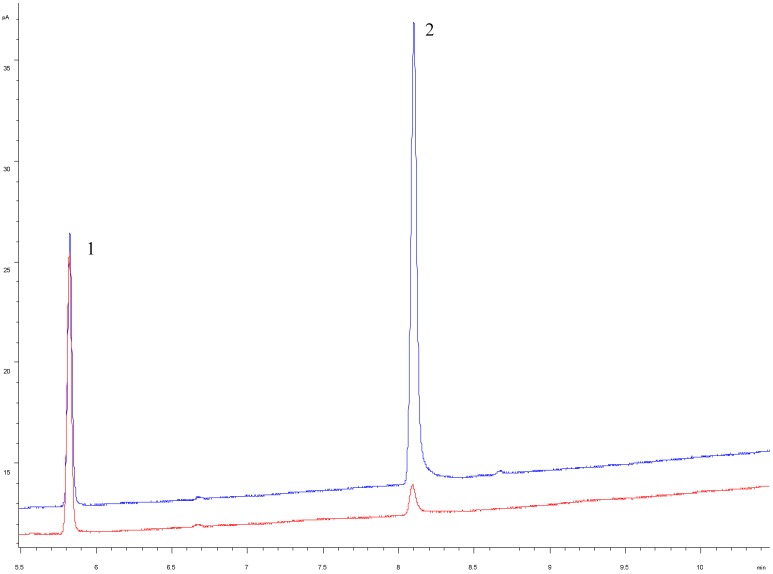
Cholesterol profile by gas chromatography with flame ionization detector in control cheese and experimental cheese with 1% of beta cyclodextrin. Peaks: 1 = 5α-cholestane; 2 = cholesterol. Blue line: control cheese (CC); Red line: experimental cheese (EC).

The chemical composition and cholesterol removal rate of control cheese (CC) without β-CD in milk and the experimental cheese (EC) with 1% of β-CD in milk are presented in Table 1. We used 1% β-CD because in previous studies we studied different concentrations of β-CD in the range (0.1 to 1%) for the elimination of cholesterol in cow’s milk fat. We found that in that study the optimal concentration to obtain cholesterol reduction higher than 90% was with a β-CD concentration approx. 0.8% [15]. Fat, moisture and protein content showed similar ratio between the CC and the EC (34.50 ± 1.12% vs. 32.51 ± 1.18%; 36.79 ± 1.65% vs. 38.15 ± 1.93%; 25.68 ± 1.04% vs. 25.10 ± 1.16%) respectively. Fat/dry matter and protein/dry matter (%) were slightly lower in EC with β-CD that the CC as a result of the higher moisture content, as suggested in the study by Seon et al. [20]. The lower fat content of the cholesterol reduced cheese than the control might be attained to the less incorporation with casein via a fat protein network, probably due to modification of the casein matrix by β-CD [25]. Soluble nitrogen (SN) and non-protein nitrogen (NPN) showed differences (*p* ≤ 0.05) between CC and EC cheese (4.76 ± 0.23% vs. 5.79 ± 0.32%; 2.41 ± 0.19% vs. 3.95 ± 0.24%), this could be due to the slight increase in the proteolysis in EC cheese that may reflect a higher peptidase activity in the EC by the influence of the β-CD [26]. During the ripening period proteolysis occurs which is an important biochemical event governing the sensory profile. The insoluble caseins are partially converted into polypeptides and amino acids. Treatment of the milk with β-CD from which cheese is manufactured results in modification of caseins matrix and thus altering the SN and NPN and consequently could be accelerate a little the ripening period of the cheese. The cholesterol removal rate of CC related to EC (195.67 ± 6.03 mg/100 g fat vs. 1.37 ± 0.19 mg/100 g fat) reached a reduction of 97.29% (Figure 1). Similar cholesterol removal were also found by Kwak et al. [27] in a study of removal of cholesterol from Cheddar cheese and Kin et al. [28] in blue cheese using β-CD. The remain β-CD showed also differences (*p* ≤ 0.05) between CC and EC with value of 0.31%. It confirms that cholesterol removal by β-CD does not affect the proximate chemical composition of Manchego ewe’s milk cheese.

### 2.2. Lipid Characteristics

Table 2 shows mean values of fatty acids (%) of CC and EC cheeses. Concentrations of individual fatty acids did not exhibit significant differences (*p* ≤ 0.05) between fat from the CC and EC cheese with β-CD. There are few reports regarding studies in manufacturing low cholesterol cheeses by β-CD and the effect on the lipidic fraction. Chen et al. [29], using supercritical fluid extraction with carbon dioxide for fractionating milk fat to remove cholesterol, observed that the fractionated milk fat showed considerable differences in fatty acids composition compared with the control cheeses. The amounts for short and medium chain fatty acids reported by these authors were 40% and 10% less, respectively, in the extracted milk fat compared with the control milk fat. Similar results were found by Gonzalez et al. [10] in a study on solubility of fatty acids in cream from ewe’s milk using supercritical fluid carbon dioxide

In our study using β-CD for removing cholesterol and the effect on the composition for short-(C4 to C8) (2.24 ± 0.19% vs. 2.14 ± 0.26%; 1.74 ± 0.06% vs. 1.68 ± 0.05%; 1.70 ± 0.05% vs. 1.66 ± 0.08%), medium-(C10 to C12) (5.02 ± 0.15% vs. 4.95 ± 0.13%; 3.19 ± 0.11% vs. 3.14 ± 0.18%), and long chain-(C14 to C18) (9.22 ± 0.84% vs. 9.21 ± 0.51%; 27.16 ± 1.52% vs. 27.41 ± 1.18%; 13.39 ± 0.55% vs. 13.59 ± 0.52%) fatty acids were no significantly different (*p* ≤ 0.05) between groups respectively. Similar results were found by Alonso et al. [15], in their study of using β-CD to decrease the level of cholesterol in milk fat.

Table 3 shows the mean values of the individual groups of triglyceride composition of fats of the CC and EC cheeses. The triglycerides of the fat cheese were resolved into 16 groups from C26 to C54. Each group is the sum of the different molecular species of triglycerides that contain the same number of carbon atoms. None of differences between control and experimental cheese with β-CD were observed (*p* ≤ 0.05), in the ∑ short-(C24–C32) (1.76 ± 0.20% vs. 1.84 ± 0.56%), ∑ medium-(C34–C48) (77.45 ± 0.85% vs. 77 ± 0.91%), and ∑ long-(C50–C54) (4.53 ± 0.39% vs. 4.43 ± 0.51%). No prior research studies have been reported on the triglycerides in cheeses treated with β-CD for removing cholesterol. Chen et al. [29], Bhaskar et al. [30], and Gonzalez et al. [10], using different techniques, found variations in triglycerides composition between control and experimental milks. The supercritical fluid extraction methods used by these investigators may have caused some variation in triglycerides composition because the triglycerides were removed by solvent extraction, that could have selectively extracted some triglycerides better than other.

In relation to the phospholipid fraction, Table 4 shows the composition in phospholipids (%) of CC and EC Manchego cheeses. Analysis of variance did not reveal any significant difference (*p* ≤ 0.05) in relative composition of the different phospholipid classes among between groups of cheeses related to the total phospholipids. Phosphatidylethanolamine (42.42 ± 4.05% vs. 38.25 ± 1.40%) was the most predominant phospholipid followed by phosphatidylcoline (27.23 ± 0.74% vs. 1.04 ± 2.21%) and sphyngomyelin (26.70 ± 5.32% vs. 25.20 ± 1.53%). Similar results were obtained by Alonso et al. [31], in a study of the effect of the β-CD on phospholipids of the milk fat in pasteurized milk. These three species of phospholipids represented more than 80% of the total phospholipids in dairy products. One of the reasons why the β-CD did not affect to these components of the milk fat could be based on the fact that β-CD specifically forms an inclusion complex with cholesterol. The central cavity of β-CD is hydrophobic, giving the molecule its affinity for non-polar molecules such as cholesterol. The radius of the cavity is such as to accommodate a cholesterol molecule almost exactly, conferring the highly specific nature of the β-CD ability to form an inclusion complex with cholesterol. They are therefore accessible to β-CD in the aqueous phase forming the insoluble inclusion complex which can be removed by centrifugation [15].

### 2.3. Flavor Characteristics

Flavor compounds isolated from CC and EC cheeses with three months of ripening are shown in Table 5. A total of 13 flavor compounds were isolated in both cheeses and some differences were observed between samples. In all cheeses, 13 flavor compound were detected, including five ketones, three aldehydes and five alcohols. Analysis of the variance did not reveal any significant difference in the total amount of ketones (2505.61 ± 36.40 ppm vs. 2314.95 ± 26.07 ppm) aldehydes (1139.63 ± 18.68 ppm vs. 1377.45 ± 24.94 ppm) and alcohols (4235.77 ± 17.13 ppm vs. 4808.87 ± 23.79 ppm) between CC and EC cheeses. 3-methylbutanal (1121.42 ± 48.32 vs. 1358.96 ± 70.32) and ethanol (4107.60 ± 62.30 ppm vs. 4685.30 ± 95.79 ppm) were the only compounds significantly different (*p* ≤ 0.05) found in CC and EC cheeses, ethanol production was the highest among flavor compounds measured, similar to those found by Kwak et al. [27] in Cheddar cheese treated with β-CD. In the study by Jeon et al. [32] of the removal of cholesterol of cream cheese by β-CD, no differences were found in the overall flavour compounds in the treated cheese compared to the regular cream cheese.

Ketones with odd carbon number have typical odor characteristics and low perception thresholds. These compounds are formed by β-oxidation and decarboxylation of fatty acids. It is known that aldehydes are not the major compounds in cheeses, as they are rapidly converted to alcohols or their corresponding acids. Branched chain aldehydes like 3-methylbutanal are formed by the catabolism of branched chain amino acids by an aminotransferase [33], and this compound was the only statistically different (*p* ≤ 0.05) in EC comparing with the CC cheese together with the ethanol. 3-Methylbutanal is an intermediate in the catabolism of leucine. Lactic acid bacteria present in the cheese together with some yeast are involved in the formation of 3-methylbutanal and alcohols (ethanol) during ripening of the cheese [33], and in our study there is a high proteolysis in the EC with a high content in non-protein nitrogen (include aminoacids as leucine), and this is the main reason why the content of 3-methylbutanal is higher in EC than in the CC. Ethanol was also higher in EC than in CC, due that this compound is also an intermediate in the catabolism of aminoacids and in the fermentation of the residual lactose by the yeast and lactic acid bacteria [32].

The amounts of short chain free fatty acids (SCFFAs), acetic, propionic, butyric and caproic acids in the control and cholesterol reduced cheeses are shown in Table 6. There was no significant difference (*P* ≤ 0.05) in total and individual amounts of FFAs (149.14 ± 5.86 ppm vs. 154.70 ± 6.12 ppm) at the end of the three month ripened, between the CC and EC cheeses. These results indicate that there was no differences in the amounts of short chain FFAs between the control and the cholesterol reduced cheese made by β-CD. Similar results in the amount of short chain SCFFAs in the control and cholesterol reduced process and cheddar cheese made by β-CD were found by [27,34]. The release of butyric and caproic acid at the three months ripening contribute to the backbone characteristics of Manchego cheese [35,36].

The sensory attributes of CC and EC cheese for a maximum of 5 score are shown in Table 7. No significant differences (*p* ≤ 0.05) were observed in flavor (3.32 ± 0.44 vs. 3.07 ± 0.89), arome (3.59 ± 0.49 vs. 3.28 ± 0.83), color (3.69 ± 0.68 vs. 3.49 ± 0.73) and acceptability (3.45 ± 0.60 vs. 3.22 ± 0.76) between CC and EC cheese. These attributes are correlated with the production of SCFFAs acids and methyl ketones during ripening (3 months) in the CC and EC cheese, which were not affected the treatment with β-CD. Texture was significantly different (*p* ≤ 0.05) in the EC with respect to the CC (3.70 ± 0.57 vs. 3.29 ± 0.72). This could be due than in the experimental cheese resulted in a higher proteolysis due to a greater peptidase activity in the cholesterol reduced cheese, that is higher in the EC by the treatment with β-CD and an slight high moisture in the cheese treated, increased by β-CD, which resulted in a slow drainage, as suggested Metzge et al. [37]. The overall preference was maintained over the ripening period of three months and no differences were found between CC and EC for flavor, aroma, color and acceptability. This study indicates that even though some differences were observed, most of the sensory characteristics and overall preferences were comparable to those of the control and three months cheese ripened treated with β-CD. Therefore, we may suggest the possibility of cholesterol reduced Manchego cheese manufactured by β-CD.

## 3. Materials and Methods

### 3.1. Chemicals

α-Cyclodextrin (α-CD), β-cyclodextrin (β-CD) and all reagents grade were supplied by Sigma (St. Louis MO, USA). Deionized water was prepared by a water purification system (Millipore Co., Burlington, MA, USA).

### 3.2. Manchego Manufacture

Ewe’s milk was previously treated with 1% β-CD by the method described by Alonso et al. [15]. One hundred L volumes of whole pasteurized milk (74 °C for 15 s) milk containing 1.0% *wt*/*vol* of β-CD were placed in a cold room at 4 °C and mixed by a stirrer (430 rcf) during 30 min. After mixing, the treated milk was left standing overnight at 4 °C (to allow time for binding the cholesterol) and precipitate the cholesterol-β-CD complex at the bottom of the tank. The upper layer without the complex was separated for making the cheeses. Manchego cheese was made by the procedure described by Fernández-García et al. [35]. Cheeses were ripened at a temperature of 12–14 °C with relative humidity of 85–90% during 3 months. The cheese-making experiment was carried out in triplicate for control and cheeses treated with 1% of β-CD.

### 3.3. Gross Composition

Fat, moisture and protein contents and nitrogen fractions were determined using the method by Alonso et al. [38].

### 3.4. Beta Cyclodextrin Analysis

β-CD was analysed by the method proposed by Alonso et al. [39]. Ten g of cheese was mixed with 5 mg of α-CD dissolved in one mL of water (internal standard for quantitative analysis). After shaking for 2 min at 40 °C it was centrifuged at room temperature for 40,000 rpm for 30 min, the upper layer was separated and filtered through a 0.45 μm membrane (Millipore Co.). A 30 μL aliquot of the supernatant spiked with the internal standard were transferred to the autosampler. A 10 μL aliquot of the supernatant was injected onto column for HPLC analysis.

The apparatus used for HPLC analysis was a Waters Alliance 2695 separation module coupled to a 410 refractive index (RI) detector, data acquisition and analysis were performed using the Empower 2 chromatography data software (Waters, Milford, MA, USA). Separation was carried out on YMC ODS-AQ column (Teknochroma, Miami, FL, USA). The mobile phase composition was a mixture of methanol and water (7:93) in isocratic condition at a flow rate 1 mL/min. The standard solutions were prepared in water to establish elution time and the quantification of β-CD was conducted by comparing sample peak area of β-CD with α-CD as the internal standard.

### 3.5. Lipid Extraction

Lipids were extracted from samples following a procedure described by an International Standard Method for milk and milk products [40]. Briefly, it consisted of an addition of an ammonia-ethanol solution to a test portion followed by lipid extraction using diethyl ether and hexane. Then, the upper layer was removed, and the solvent completely evaporated. The lipid extracts obtained were placed into amber glass vials, flushed with a stream of nitrogen and stored at −20 °C until analyzed.

### 3.6. Determination of Cholesterol

The technique chosen for cholesterol determination was as described by Alonso et al. [41] using direct injection of milk fat by capillary gas chromatography (GC). Approximately 30 mg anhydrous milk fat and 0.1 mL 5-α-cholestane as internal standard (3.5 mg/mL in hexane) was dissolved in 1 mL of hexane; 0.5 μL of the resulting solution was injected for GC analysis. For GC analysis for free cholesterol by this direct method we used an Agilent Technology 6890 chromatograph (Palo Alto, CA, USA) equipped with flame ionization detector. Analyses were performed using a HP-5 fused silica capillary column (30 m × 0.32 mm i.d. 0.25 μm thickness). Experimental chromatographic conditions were: He carrier gas at 17 psi head pressure; initial column temperature 280 °C, held for 1 min, increased to 355 °C at 3 °C/min. Injector temperature 350 °C and detector temperature was 360 °C. Peak identification was done by comparison of relative retention times with retention times of standards. Quantification of cholesterol was conducted by comparing sample peak area with of the 5 α-cholestane internal standard. The percentage of cholesterol reduction in milk fat was calculated by the formula [(100 − amount of cholesterol in milk fat) × 100]/amount of cholesterol in untreated milk).

### 3.7. Fatty Acids and Triglycerides Analysis

Fatty acids methyl esters (FAMES) were prepared by alkaline catalyzed methanolysis of the extracted lipids using 2 N KOH in methanol. The FAMES were analyzed on an Agilent Technology 6890 chromatograph (Palo Alto, CA, USA) with FID detector. Fatty acids were separated using CP-Sil 88 fused-silica capillary column (50 m × 0.25 mm i.d. × 0.2 μm film thickness, Chrompack, CA, USA) using the method described by Alonso et al. [42]. GC analysis of triglycerides by direct injection was performed on an Agilent gas chromatograph 6890 (Palo Alto, CA, USA) equipped with flame ionization detector. Analyses were performed using a WCOT fused silica capillary column (25 m × 0.25 mm × 0.1 μm film thickness) coated with OV 17 TRI (J.W. Scientific, Polson, CA, USA) using the method described by Alonso [43].

### 3.8. Phospholipids Analysis

Extractions of cheese fat were carried out with an Accelerated Solid Extraction ASE-200 extractor (Dionex Corp., Sunnyvale, CA, USA) using 2 g of freeze-dried cheese sample that was well mixed with 2 g of sea sand and loaded into a stainless steel extraction cell covered with filters on both sides. For the maximum cheese fat yield, the extraction included the use of dichloromethane-methanol solution (2:1, *vol*/*vol*) as solvent mixture and 10.3 MPa of pressure as fixed conditions described by Castro-Gómez et al. [44].

Separation of lipid classes was accomplished in an HPLC system (model 1260; Agilent Technologies Inc., Santa Clara, CA, USA) coupled with an evaporative light scattering detector (SEDEX 85 model; Sedere SAS, Alfortville CEDEX, France) using prefiltered compressed air as the nebulizing gas at a pressure of 350 kPa at 60 °C; the gain was set at 3. Two columns in series (250 × 4.5 mm Zorbax Rx-SIL column with 5-μm particle diameter; Agilent Technologies Inc.) and a precolumn with the same packing were used [44].

### 3.9. Analysis of Volatile Compounds

Analysis of volatile fraction was performed by headspace gas chromatographic mass spectrometric (GC-MS) method described by Alonso et al. [45]. To 10 g of previously homogenized cheese, 80 μL of aqueous solution of propionic acid ethyl methyl ester (1.14 mg/mL) as internal standard and anhydrous sodium sulphate (10 g) to retain water 176 were added. Individual standard dilutions in aqueous solution were prepared and were stored hermetically in sealed vials at 20 °C until their use. Prior to be analyzed in a static headspace apparatus (Model HSS 19395; Hewlett Packard), the samples were maintained at 80 °C for 60 min until the sample and gaseous phase reached the thermodynamic equilibrium. Apparatus was programmed as follows: 5 s pressurization, 18 equilibrium and filling and 2 min for injection. Helium was employed as carrier gas at a flow 18 rate of 17.5 mL/min. A Hewlett Packard GC Model 5890 coupled to selective MS Model 5972 was employed for volatile compounds analysis. Samples were injected in the split mode (split 18 rate of 7:1) on a capillary silica column with polyethylene glycol (HP Innovas, 60 m, 0.25 mm 18 ID, 0.25 μm film thickness, Hewlett Packard). Helium was used as carrier gas, at a flow rate of 18 36.5 cm/s. The column temperature program was: 33 °C for 5 min, increase at 1 °C/min up to 38 °C and then at 7 °C/min up to 210 °C, and held for 10 min. Injection was carried out at 200 °C and the interface line of MS at 280 °C. Electronic ionisation energy and photomultiplier voltage 18 were 70 eV and 1647 V, respectively.

### 3.10. Short Chain Free Fatty Acids

For the analysis of SCFFAs, cheese sample (1 g) was homogenized in 20 mL of distilled water, centrifuge at 10,000 rpm for 10 min and filtered by 0.40 μm filter. A Hewlett-Packard model 5890 A equipped with a flame ionization detector on a capillary silica column (HP FFAP, 30 m, 0.25 mm ID, 0.25 μm film thickness, Agilent J & W) was used for analysis. Quantitative analysis were done using 2-ethylbutanoic acid as internal standard.

### 3.11. Sensory Analysis

Samples of Manchego cheese were cut in slices of approximately 8 × 8 cm of a thickness of approx. 1 cm and placed on white plates. Samples were tempered at ambient temperature (20 ± 2 °C) and then presented to the panelists. Twenty two trained sensory panelists from the members of the research Institute which, trained in sensory analysis of cheese, evaluated randomly coded cheeses. The testing conditions of the room for the sensory analysis were in conformity with the ISO requirements [46]. Flavor, aroma, color, texture and acceptability were evaluated on a five point scale (1 = poor to 5 = excellent).

### 3.12. Statistical Analysis

Experimental data were treated by analysis of variance (ANOVA) using the statistical software SAS (version 8.02, SAS Institute Inc., Cary, NC, USA). Differences among treatments were determined by statistical analysis using a Student t-test where *p* ≤ 0.05 was considered statiscally significant.

## 4. Conclusions

Approximately 97.6% cholesterol reduction was observed in the cheese that was treated using β-CD. Physicochemical properties (fat, moisture and protein) were not changed by the β-CD treatment, except the NS and NNP that showed slight differences attributed to the treatment. The amount of the different components of the lipid fraction (fatty acids, triglycerides and phospholipids) were similar in both, treated and untreated cheese with β-CD. Flavor compounds and short chain free fatty acids were mostly not significantly influenced by the β-CD. Although, the β-CD molecules are edible and nontoxic and a results they can be used safely for cholesterol removal processing. Therefore, the present study suggested that the treatment with the β-CD was an effective process for cholesterol removal from Manchego cheese, while preserving its nutritional properties. Further studies to evaluate the effect of the intake of the control and low cholesterol Manchego cheeses on the concentration of serum cholesterol would be of interest.

## Figures and Tables

**Table 1:** Gross composition of the control and the experimental Manchego cheese by the effect of the β-CD.

Parameter	CC	EC	REE
Fat (%)	34.50 ± 1.12 ^a^	32.51 ± 1.18 ^a^	5.77
Moisture (%)	36.79 ± 1.65 ^a^	38.15 ± 1.93 ^a^	3.70
Protein (%)	25.68 ± 1.04 ^a^	25.10 ± 1.16 ^a^	8.77
SN (% as protein)	4.76 ± 0.23 ^a^	5.79 ± 0.32 ^b^	2.26
NPN (% as protein)	2.41 ± 0.19 ^a^	3.95 ± 0.24 ^b^	6.39
pH	4.87 ± 0.15 ^a^	4.85 ± 0.25 ^a^	0.41
Cholesterol (mg/100 g fat)	195.67 ± 6.03 ^a^	4.72 ± 0.19 ^b^	99.30
Cholesterol removal (% fat)	-	97.6 ± 4.56	-
Remain β-CD (%)	-	0.31 ± 0.13	-

CC, control cheese without β-CD in milk; EC, experimental cheese with 1% β-CD in milk; SN, soluble nitrogen (% as protein); NNP, non-protein nitrogen (% as protein); REE (%), relative experimental error; Mean standard deviation (*n* = 12); ^a,b^ Different letters in the same row mean significant differences (*p* ≤ 0.05).

**Table 2:** Fatty acids composition (g/100 g fat) from the control and the experimental Manchego cheese by the effect of β-CD.

Fatty Acid	CC	EC	REE
C4:0	2.24 ± 0.19 ^a^	2.14 ± 0.26 ^a^	4.46
C6:0	1.74 ± 0.06 ^a^	1.68 ± 0.05 ^a^	3.45
C8:0	1.70 ± 0.05 ^a^	1.66 ± 0.08 ^a^	2.35
C10:0	5.02 ± 0.15 ^a^	4.95 ± 0.13 ^a^	1.39
C10:1	0.28 ± 0.03 ^a^	0.26 ± 0.07 ^a^	7.14
C12:0	3.19 ± 0.11 ^a^	3.14 ± 0.18 ^a^	1.57
C14.0	9.22 ± 0.84 ^a^	9.21 ± 0.51 ^a^	0.11
C14:1	0.90 ± 0.03 ^a^	0.86 ± 0.06 ^a^	4.44
C15:0	0.24 ± 0.02 ^a^	0.25 ± 0.05 ^a^	4.17
C16:0	27.16 ± 1.52 ^a^	27.41 ± 1.18 ^a^	0.92
C16:1	0.73 ± 0.12 ^a^	0.77 ± 0.17 ^a^	5.48
C17:0	0.54 ± 0.07 ^a^	0.58 ± 0.07 ^a^	7.41
C18:0	13.39 ± 0.55 ^a^	13.59 ± 0.52 ^a^	1.49
C18:1t	2.62 ± 1.13 ^a^	2.65 ± 0.23 ^a^	1.15
C18:1c	23.28 ± 0.35 ^a^	22.93 ± 1.16 ^a^	1.50
C18:2	3.9 ± 0.08 ^a^	3.66 ± 0.24 ^a^	6.15
C18:3	0.39 ± 0.08 ^a^	0.40 ± 0.05 ^a^	2.56
C18:2 (c9t11)	0.96 ± 0.06 ^a^	0.97 ± 0.06 ^a^	1.04

CC, control cheese without β-CD in milk; EC, experimental cheese with 1% β-CD in milk; REE (%), relative experimental error; Mean standard deviation (*n* = 12); ^a^ Different letters in the same row mean significant differences (*p* ≤ 0.05).

**Table 3:** Triglycerides composition (g/100 g fat) from the control and the experimental Manchego cheese by the effect of the β-CD.

Triglyceride	CC	EC	REE
C24	0.33 ± 0.06 ^a^	0.32 ± 0.08 ^a^	3.03
C26	0.88 ± 0.09 ^a^	0.80 ± 0.05 ^a^	9.09
C28	1.64 ± 0.15 ^a^	1.54 ± 0.13 ^a^	6.09
C30	2.42 ± 0.23 ^a^	2.47 ± 0.21 ^a^	2.07
C32	3.54 ± 0.40 ^a^	3.25 ± 0.39 ^b^	8.59
C34	4.89 ± 0.38 ^a^	5.04 ± 0.48 ^a^	3.07
C36	7.21 ± 0.66 ^a^	7.04 ± 0.54 ^a^	2.36
C38	10.66 ± 1.11 ^a^	10.65 ± 1.30 ^a^	0.09
C40	17.35 ± 1.32 ^a^	17.89 ± 1.32 ^a^	3.11
C42	16.02 ± 1.40 ^a^	16.17 ± 1.50 ^a^	0.94
C44	8.83 ± 0.77 ^a^	8.13 ± 0.66 ^a^	8.13
C46	7.14 ± 0.62 ^a^	7.04 ± 0.52 ^a^	1.40
C48	5.35 ± 0.55 ^a^	5.71 ± 0.49 ^a^	6.73
C50	4.28 ± 0.35 ^a^	4.39 ± 0.51 ^a^	2.57
C52	4.57 ± 0.39 ^a^	4.31 ± 0.56 ^a^	5.69
C54	4.78 ± 0.43 ^a^	4.58 ± 0.45 ^a^	4.18

CC, control cheese without β-CD in milk; EC, experimental cheese with 1% β-CD in milk; REE (%), relative experimental error. Mean standard deviation (*n* = 12); ^a,b^ Different letters in the same row mean significant differences (*p* ≤ 0.05).

**Table 4:** Phospholipids composition of the control and the experimental Manchego cheese by the effect of the β-CD.

Phospholipids	CC	EC	REE
Total PLs (mg/100 g fat)	0.12 ± 0.03 ^a^	0.11 ± 0.03 ^a^	8.83
PE (% of PL)	42.42 ± 4.05 ^a^	38.25 ± 1.40 ^a^	9.83
PI (% of PL)	1.93 ± 1.31 ^a^	2.46 ± 0.62 ^a^	0.27
PS (% of PL)	1.75 ± 0.53 ^a^	3.21 ± 1.94 ^a^	1.20
PC (% of PL)	27.23 ± 0.74 ^a^	31.04 ± 2.21 ^a^	0.14
SM (% of PL)	26.70 ± 5.32 ^a^	25.20 ± 1.53 ^a^	0.06

CC, control cheese without β-CD in milk; EC, experimental cheese with 1% β-CD in milk; PLs, Phospholipids; PE, phosphatidylethanolamine; PI, phosphatidylinositol; PS, phosphatidylserin; PC, phosphatidylcoline; SM, sphyngomyelin; REE (%), relative experimental error; Mean standard deviation (*n* = 12); ^a^ Different letters in the same row mean significant differences (*p* ≤ 0.05).

**Table 5:** Volatile compounds (ppm) of the control and the experimental Manchego cheese by the effect of the β-CD.

Compounds	CC	EC	REE
Ketones			
2-Propanone	420.38 ± 32.39 ^a^	381.05 ± 26.89 ^a^	9.03
2-Butanone	27.65 ± 4.51 ^a^	25.16 ± 4.21 ^a^	9.01
2,3-Butanedione	1271.54 ± 48.45 ^a^	1145.81 ± 56.38 ^a^	9.89
2-Heptanone	562.30 ± 29.49 ^a^	512.18 ± 22.78 ^a^	8.91
3-Hydroxy-2-butanone	186.12 ± 18.66 ^a^	248.70 ± 20.09 ^a^	3.34
Aldehydes			
3-Methylbutanal	1121.42 ± 48.32 ^a^	1358. 96 ± 70.32 ^b^	21.18
Hexanal	14.16 ± 6.50 ^a^	13.54 ± 4.09 ^a^	4.38
Nonanal	4.55 ± 1.21 ^a^	4.95 ± 1.19 ^a^	8.79
Alcohols			
2-Propanol	13.50 ± 3.56 ^a^	12.64 ± 3.70 ^a^	6.31
Ethanol	4107.60 ± 62.30 ^a^	4685.30 ± 95.79 ^b^	14.07
2-Methyl-1-propanol	49.18 ± 7.11 ^a^	45.66 ± 7.80 ^a^	7.16
2-Butanol	29.31 ± 6.85 ^a^	26.69 ± 5.56 ^a^	8.94
2-Heptanol	36.18 ± 6.04 ^a^	39.57 ± 6.12 ^a^	9.37

CC, control cheese without β-CD in milk; EC, experimental cheese with 1% β-CD in milk; REE (%), relative experimental error; Mean standard deviation (*n* = 12); ^a,b^ Different letters in the same row mean significant differences (*p* ≤ 0.05).

**Table 6:** Short chain free fatty acids (SCFFA) (ppm) of the control and the experimental Manchego cheese by the effect of the β-CD.

SCFFA	CC	EC	REE
Acetic	92.91 ± 7.19 ^a^	95.06 ± 6.19 ^a^	2.31
Propionic	35.28 ± 5.65 ^a^	38.36 ± 4.96 ^a^	8.73
Butyric	17.10 ± 3.96 ^a^	17.32 ± 3.60 ^a^	1.29
Caproic	13.85 ± 2.52 ^a^	13.96 ± 3.12 ^a^	0.79
Total	159.14 ± 5.86 ^a^	164.70 ± 6.12 ^a^	3.49

CC, control cheese without β-CD in milk; EC, experimental cheese with 1% β-CD in milk; REE (%), relative experimental error; Mean standard deviation (*n* = 12); ^a^ Different letters in the same row mean significant differences (*p* ≤ 0.05).

**Table 7:** Sensory analysis of the control and the experimental Manchego cheese by the effect of the β-CD. Flavor, arome, color, texture and acceptability were evaluated on a five point scale (1 = poor to 5 = excellent).

Attribute	CC	EC	REE
Flavor	3.32 ± 0.44 ^a^	3.07 ± 0.89 ^a^	7.53
Aroma	3.59 ± 0.49 ^a^	3.28 ± 0.83 ^a^	8.63
Color	3.69 ± 0.68 ^a^	3.49 ± 0.73 ^a^	5.42
Texture	3.70 ± 0.57 ^a^	3.29 ± 0.72 ^b^	11.12
Acceptability	3.45 ± 0.60 ^a^	3.22 ± 0.76 ^a^	6.65

CC, control cheese without β-CD in milk; EC, experimental cheese with 1% β-CD in milk; REE (%), relative experimental error; Mean standard deviation (*n* = 12); ^a,b^ Different letters in the same row mean significant differences (*p* ≤ 0.05).
